# p53 directly activates *cystatin D/CST5* to mediate mesenchymal-epithelial transition: a possible link to tumor suppression by vitamin D3

**DOI:** 10.18632/oncotarget.4683

**Published:** 2015-06-28

**Authors:** Sabine Hünten, Heiko Hermeking

**Affiliations:** ^1^ Experimental and Molecular Pathology, Institute of Pathology, Ludwig-Maximilians-Universität München, Munich, Germany; ^2^ German Cancer Consortium (DKTK), Heidelberg, Germany; ^3^ German Cancer Research Center (DKFZ), Heidelberg, Germany

**Keywords:** p53, CST5, vitamin D3, SNAIL, mesenchymal-epithelial transition

## Abstract

*Cystatin D (CST5)* encodes an inhibitor of cysteine proteases of the cathepsin family and is directly induced by the vitamin D receptor (VDR). Interestingly, vitamin D3 exerts tumor suppressive effects in a variety of tumor types. In colorectal cancer (CRC) cells CST5 was shown to mediate mesenchymal-epithelial transition (MET). We recently performed an integrated genomic and proteomic screen to identify targets of the p53 tumor suppressor in CRC cells. Thereby, we identified *CST5* as a putative p53 target gene. Here, we validated and characterized *CST5* as a direct p53 target gene. After activation of a conditional *p53* allele, CST5 was upregulated on mRNA and protein levels. Treatment with nutlin-3a or etoposide induced *CST5* in a p53-dependent manner. These regulations were direct, since ectopic and endogenous p53 occupied a conserved binding site in the *CST5* promoter region. In addition, treatment with calcitriol, the active vitamin D3 metabolite, and simultaneous activation of p53 resulted in enhanced *CST5* induction and increased repression of SNAIL, an epithelial-mesenchymal transition (EMT) inducing transcription factor. Furthermore, *CST5* inactivation decreased p53-induced mesenchymal-epithelial transition (MET) as evidenced by decreased inhibition of SNAIL and of migration by p53. Furthermore, *CST5* expression was directly repressed by SNAIL. In summary, these results imply *CST5* as an important mediator of tumor suppression by p53 in colorectal cancer. In addition, they suggest that a combined treatment activating p53 and the vitamin D3 pathway may function via induction of CST5.

## INTRODUCTION

During the last years the role of vitamin D, primarily vitamin D3, in tumor suppression has attracted considerable attention (reviewed in [1-4]). There is now increasing evidence strongly suggesting that vitamin D3 reduces the risk of developing cancer and may be applied for the treatment of cancer as it was shown to induce differentiation and apoptosis, and inhibit proliferation and angiogenesis (reviewed in [5]). A tumor suppressive effect of vitamin D3 has also been shown *in vivo* in animal models of colon, breast and prostate cancer [6-11]. In addition, several epidemiological studies have implicated vitamin D3 in reducing cancer risk, progression and mortality with promising results especially in colon cancer [12-20]. Several large-scale clinical trials aiming to determine the therapeutic value of vitamin D3 are expected to be finalized within the next five years [21].

Vitamin D3, the precursor of the hormonally active calcitriol, is mainly synthesized in the skin after exposure to sunlight but is also supplied by nutritional sources [22]. Two hydroxylation steps mediated by the hydroxylases CYP27A1 and CYP27B1 in the liver and in the kidney, respectively, lead to the active vitamin D3 metabolite calcitriol (1,25(OH)_2_D_3_) [23]. Interestingly, CYP27B1 is also expressed at extra-renal sites including brain, colon, pancreas or skin and mediates local conversion of the intermediate metabolite 25-hydroxy-vitamin D_3_ (25(OH)D_3_) to calcitriol [24]. Calcitriol binds to the vitamin D receptor (VDR), which regulates the expression of several genes involved in colorectal cancer, such as *BIRC5*, *CDKN1A*, *CDH1* or *HIF1α* (reviewed in [5]). Recently, calcitriol-induced expression of cystatin D (*CST5*) was shown to inhibit proliferation, migration, anchorage-independent growth as well as tumor formation of xenografted colorectal cancer cell lines [25]. Moreover, poor differentiation of colorectal tumors correlates with decreased *CST5* expression [25].

Cystatin D is an endogenous inhibitor of cystein proteases, such as the cathepsins S, H and L [26, 27]. Cysteine cathepsins are lysosomal proteases and are often upregulated in different types of cancers. During tumorigenesis cathepsins are translocated from intracellular compartments, typically lysosomes, to the outer cell-membrane or secreted into the extracellular space. Cathepsins promote tumor progression via degradation of components of the basement membrane, extracellular matrix or cleavage of the adhesion molecule E-cadherin (reviewed in [28]).

The p53 transcription factor is one of the most important tumor suppressors as also evidenced by its common inactivation in human tumors [29]. Several different stress signals including oncogene activation, oxidative stress or DNA damage lead to its posttranslational stabilization and subsequent association with binding sites within promoters [30], which are composed of two half sites of RRRCWWGYYY (R: purine, W: adenine or thymine, Y: pyrimidine) divided by a spacer of 0 - 13 bp [31, 32]. Once activated, p53 exerts diverse tumor suppressive effects through a large number of target genes [33]. Interestingly, p53 directly induces the expression of the VDR [34, 35]. Moreover, the *CDKN1A* promoter harbors p53 and VDR binding sites suggesting the possibility of coordinated regulations after p53 and VDR activation [36]. Furthermore, calcitriol induces MDM2 expression in a p53-dependent manner therefore limiting p53 activation [37]. In addition, mutant p53 may convert the effect of vitamin D3 by modulating VDR-mediated transcription [38].

Here, we show that p53 directly induces *CST5* expression by binding to a p53 response element upstream of the *CST5* promoter. We present evidence that a combination of p53 activation and calcitriol treatment results in enhanced *CST5* expression and suppression of the EMT transcription factor *SNAIL*. Furthermore, we show that upregulation of *CST5* by p53 contributes to the induction of mesenchymal-epithelial transition. In addition, we demonstrate that SNAIL directly represses *CST5* expression and that calcitriol treatment reverses this effect.

## RESULTS

### p53 induces *CST5* expression

**Figure 1:**
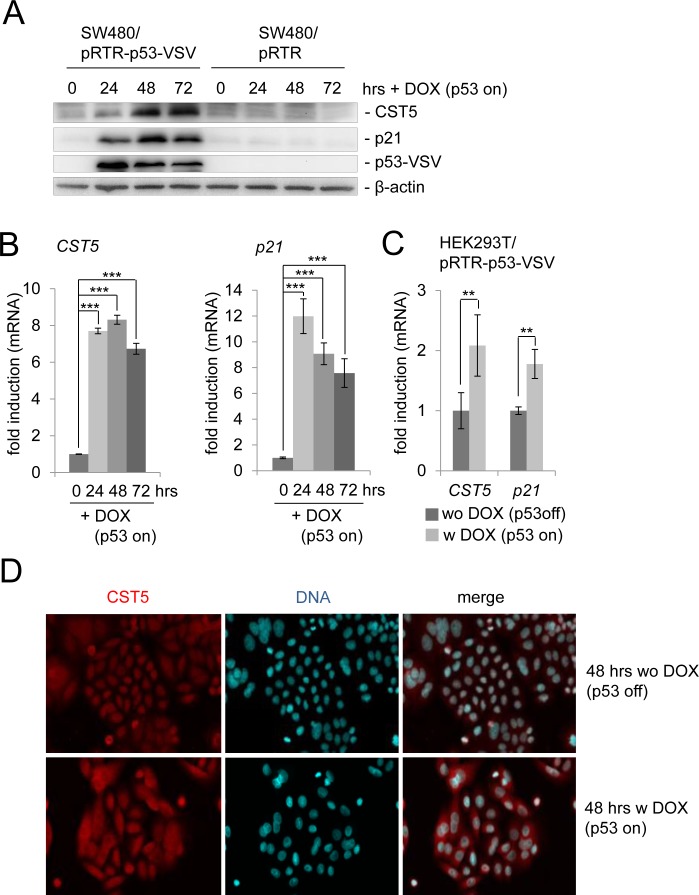
Regulation of CST5 by p53 in SW480 colorectal cancer cells SW480 cells harboring a pRTR-p53-VSV vector or the empty vector pRTR were treated with DOX for the indicated periods of time to activate p53-VSV expression. **A.** Western blot analysis of the indicated proteins in SW480/pRTR-p53-VSV. β-actin served as a loading control. **B.** The expression of the *CST5*- and *p21*-mRNA was determined by qPCR analysis. Fold changes represent mean values of triplicate analyses of DOX-versus un-treated cells normalized to *β-actin* expression. Standard deviations are represented by error bars. **C.** HEK293T cells were transfected with the pRTR-p53-VSV vector and p53 expression was induced by adding DOX for 24 hours. The expression of *CST5-* and *p21*-mRNA levels was measured by qPCR analysis. Fold changes represent mean values of triplicate analyses of DOX-treatment versus untreated cells normalized to *β-actin* expression. Standard deviations are represented by error bars. **D.** Analysis of CST5 expression and localization in SW480/pRTR-p53-VSV cells by confocal immunofluorescence microscopy after 48 hours with and without DOX treatment. Nuclear DNA was visualized by DAPI staining. 200x magnification.

We had previously observed a ~6-fold increase of CST5 protein by pulsed SILAC and a ~9-fold induction of *CST5* mRNA expression by RNA-Seq analysis after addition of doxycycline (DOX) to activate expression of ectopic p53 in the colorectal cancer cell line SW480 harboring a pRTR-p53-VSV vector for 48 and 40 hours, respectively (Hünten et al., submitted). Here, we confirmed the upregulation of the CST5 protein by p53 by Western blot analysis (Figure 1A). As expected, p53 expression also resulted in the induction of the known p53 target p21. The kinetics of induction by p53 were similar for p21 and CST5: at 24 hours an induction was detectable and by 72 hours both proteins were still elevated. After p53 activation, *CST5* mRNA expression displayed a similar induction as *p21* (Figure 1B). Recently, *CST5* was shown to be induced by vitamin D3 via direct binding of the vitamin D receptor (VDR) to its promoter in human colon cancer cell lines [25]. In addition, the *VDR* gene is a known p53 target [34]. To determine whether p53 induces *CST5* in a VDR-independent manner, VDR-deficient HEK293T cells were transfected with a DOX-inducible pRTR-p53-VSV vector (Figure 1C). Activation of p53 expression by treatment with DOX resulted in a significant induction of *CST5* and, as a control, *p21* mRNA. These results show that p53 induces *CST5* expression in a VDR-independent manner. An immunofluorescence analysis confirmed increased CST5 protein levels after ectopic p53 expression and showed its predominant cytoplasmic localization after DOX treatment in SW480/pRTR-p53-VSV cells (Figure 1D).

### Endogenous p53 mediates induction of *CST5* by DNA damage

**Figure 2:**
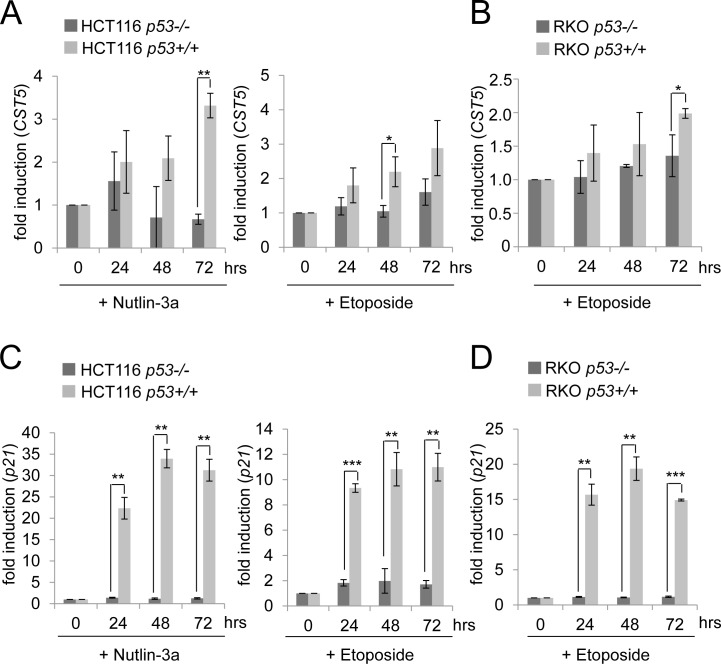
p53-dependent regulation of *CST5* in HCT116 and RKO cancer cells **A.**, **B.** *CST5*- and **C.**, **D.** *p21*-mRNA levels were measured by qPCR analysis at the indicated time points. **A.**, **C.** HCT116 *p53−/−* or *p53+/+* and **B.**, **D.** RKO *p53+/+* or *p53−/−* were treated with nutlin-3a or etoposide or the vehicle DMSO. Fold changes represent mean values of triplicate analyses of nutlin-3a/etoposide versus DMSO treated cells normalized to *β-actin* expression. Error bars represent standard deviations.

To further interrogate the p53-dependency of the *CST5* expression, the colorectal cancer cell lines HCT116 and RKO and their isogenic p53-deficient variants, which were generated by homologous recombination [39], were treated with either nutlin-3a, a small-molecule inhibitor of MDM2 [40], or the DNA-damaging agent etoposide (Figure 2). Both treatments resulted in a time-dependent upregulation of *CST5* in HCT116 *p53+/+* cells (Figure 2A). Since *CST5* expression was not induced in HCT116 *p53−/−* cells, the activation of *CST5* transcription was mediated by p53. Also RKO cells showed a p53-dependent induction of *CST5* after induction of DNA damage by etoposide (Figure 2B). The p53-dependent induction of *p21* mRNA was more pronounced than the induction of *CST5* expression in RKO and HCT116 cells (Figure 2C, 2D). Taken together, these results show that p53 mediates the induction of CST5 by DNA damage.

### *CST5* is a direct p53 target

**Figure 3:**
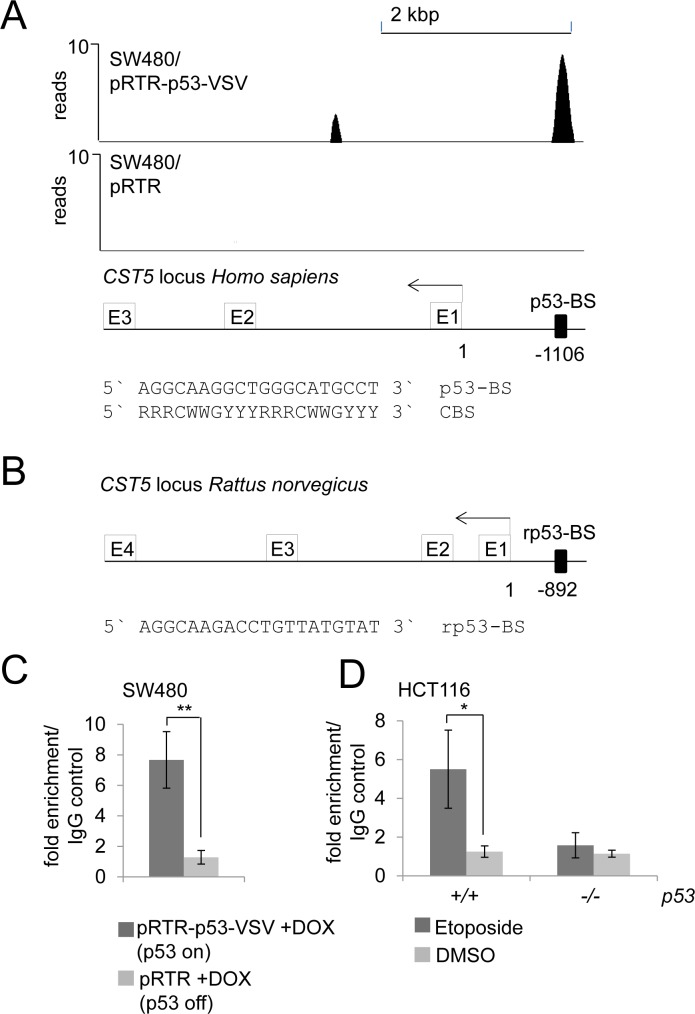
Direct binding of p53 upstream of the *CST5* promoter **A.** ChIP-Seq analysis was performed with a VSV-specific antibody 16 hours after addition of DOX to SW480/pRTR-p53-VSV and SW480/pRTR cells. The ChIP-Seq results are represented in the UCSC browser showing p53 binding to the indicated p53 binding site in a region 1106 bp upstream of the *CST5* promoter. **B.** p53 binding site upstream of the *CST*5 locus of *Rattus norvegicus*. **C.** qChIP analysis of SW480/pRTR-p53-VSV cells 16 hours after activation of p53-VSV expression versus SW480/pRTR cells using anti-VSV and anti-rabbit IgG for ChIP. Experiments were performed in triplicates. Error bars represent +/− SD (*n* = 3). **D.** qChIP analysis of HCT116 *p53+/+* versus HCT116 *p53−/−* after 16 hours etoposide treatment using anti-p53 and anti-mouse IgG for ChIP; this ChIP analysis was performed in unicates and measured in triplicates.

In a genome-wide p53/ChIP-Seq analysis we had detected ChIP-signals in the vicinity of the *CST5* promoter indicating direct p53 binding (Figure 3A; Hünten et al., submitted). A sequence that fits well to the p53 binding consensus sequence [31, 32] was identified 1106 base-pairs upstream of the transcriptional start site (TSS) of *CST5* underneath a ChIP-signal with ~10 reads (Figure 3A). This p53 binding element was conserved between human and rat *CST5* promoters (Figure 3B). p53 binding at this site was confirmed by qChIP analysis in SW480/pRTR-p53-VSV cells treated with DOX for 16 hours, whereas no enrichment was detected in the control cell line SW480/pRTR (Figure 3C). After DNA damage induced by etoposide, HCT116 *p53+/+*, but not HCT116 *p53−/−* cells, displayed increased p53 occupancy at this site (Figure 3D). Therefore, also endogenous p53 occupied these binding sites. Together with the results presented above, these findings establish *CST5* as a direct transcriptional target of p53.

### Role of CST5 in p53-mediated MET

**Figure 4:**
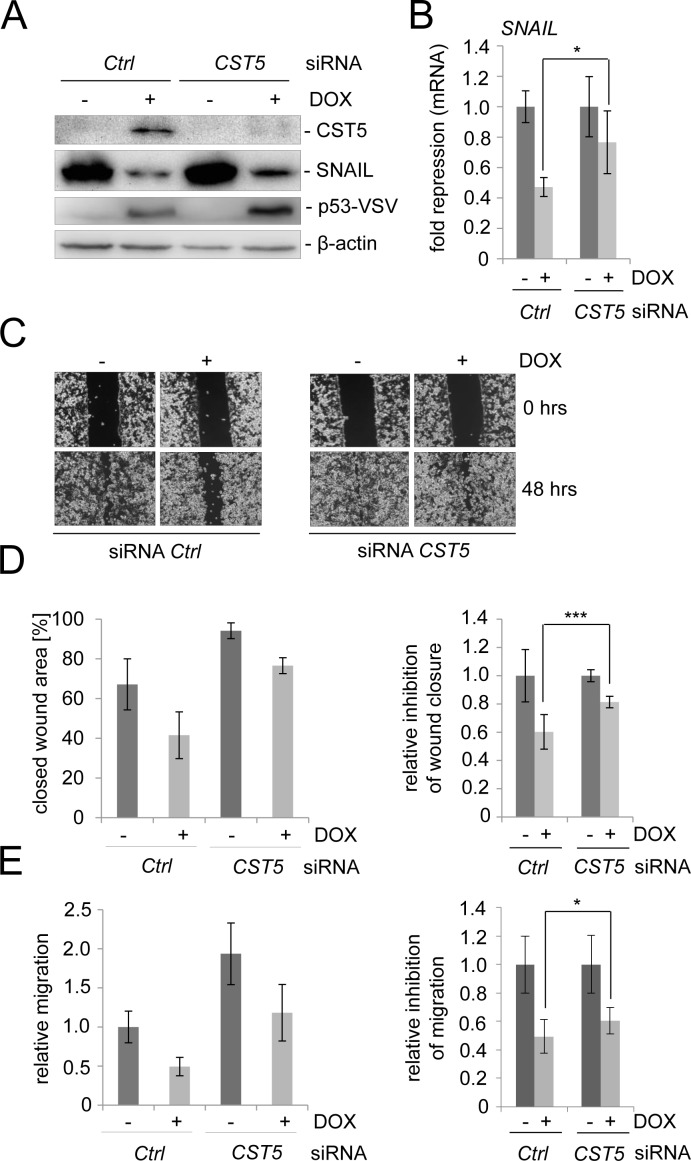
CST5 is relevant for p53-mediated MET SW480/pRTR-p53-VSV cells were transfected with a *CST5*-specific siRNA or control oligonucleotide for 72 hours and treated with DOX for 48 hours. **A.** Western Blot analysis of the indicated proteins. β-actin served as a loading control. **B.** *SNAIL* mRNA levels were measured by qPCR analysis after treatment with DOX. Fold changes represent mean values of triplicate analyses of DOX treatment versus vehicle treated cells normalized to *β-actin* expression. The relative p53-mediated repression of *SNAIL* is indicated. **C.**, **D.** Wound-healing assay using Ibidi-Inlets. **C.** Representative pictures of the wound area obtained 48 hours after scratching. 100 x magnification. **D.** Left: Average [%] of the closed wound area determined by the final width of the scratch in three independent wells. Right: The relative p53-mediated inhibition of wound-closure. **E.** Boyden chamber-assay of cellular migration. Left: The relative migration through the filter with the untreated control set as one. Right: The relative p53-mediated inhibition of migration. B, D, E: Error bars represent +/− SD (*n* = 3).

In order to determine the requirement of CST5 for p53-mediated mesenchymal-epithelial transition (MET) and related processes, such as cellular migration, we silenced *CST5* expression in the colorectal cancer cell line SW480/pRTR-p53-VSV using siRNAs (Figure 4A). After treatment with a *CST5*-directed siRNA CST5 protein was undetectable after induction of ectopic p53 expression (Figure 4A). In the presence of *CST5* silencing the p53-mediated repression of SNAIL protein was less pronounced than in cells transfected with control siRNAs (Figure 4A). The same effect was observed on the level of mRNA expression (Figure 4B). Subsequently, we determined whether downregulation of *CST5* influences p53-mediated inhibition of cellular migration in a scratch/wound-closure assay and in a Boyden-chamber assay (Figure 4C-4E). When *CST5* was silenced by siRNAs, wound closure of SW480/pRTR-p53-VSV cells was generally less suppressed by p53 than in control cells (Figure 4C, 4D). Also in a Boyden-chamber assay a reduction in the inhibition of migration by p53 after treatment with si*CST5* was observed (Figure 4E). Taken together, these results show that the induction of CST5 by p53 contributes to the inhibition of migration by p53.

### Cooperation between p53 and VDR at the *CST5* promoter

**Figure 5:**
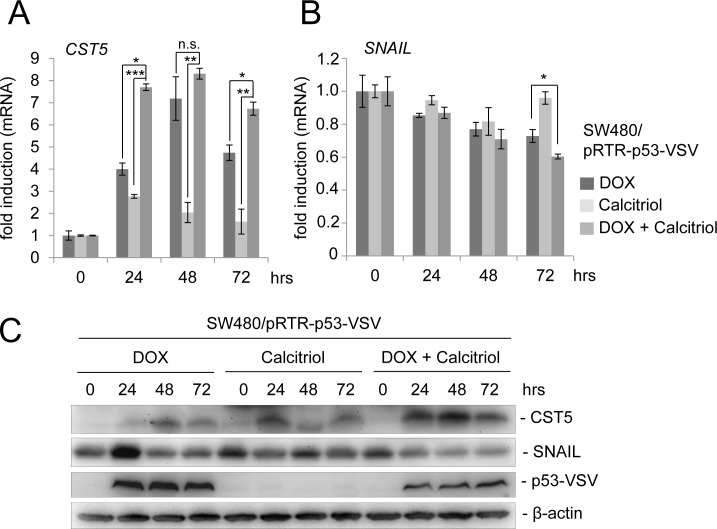
p53 activation in combination with calcitriol treatment enhances CST5 induction **A.** *CST5*- and **B.** *SNAIL* mRNA levels were measured by qPCR analysis after treatment with DOX, calcitriol or both agents at the indicated time-points. Fold changes represent mean values of triplicate analyses of DOX / calcitriol / DOX+calcitriol treatment versus vehicle treated cells normalized to *β-actin* expression. Error bars represent standard deviations. **C.** Western blot analysis of the indicated proteins in SW480/pRTR-p53-VSV cells. β-actin served as a loading control.

Recently, the active metabolite of vitamin D3, calcitriol (1,25(OH)_2_D_3_), was shown to induce *CST5* expression in colorectal cancer cells [25]. To examine whether a combination of p53 activation and treatment with calcitriol may lead to an enhanced *CST5* induction, we treated the colorectal cancer cell line SW480/pRTR-p53-VSV with DOX, calcitriol or both agents (Figure 5). As shown before, induction of ectopic p53 expression led to a robust increase in *CST5* expression on the mRNA level (Figure 5A). Calcitriol treatment alone resulted in a comparatively minor induction in *CST5* expression, whereas the combination of DOX and calcitriol led to significantly higher *CST5* mRNA levels when compared to single DOX or calcitriol treatments. Furthermore, CST5 protein induction was more pronounced after combined activation of p53 and the VDR when compared to addition of DOX or calcitriol alone (Figure 5C). In addition, the EMT transcription factor SNAIL displayed a stronger decrease on the mRNA and protein level after a combined treatment with DOX and calcitriol when compared to each stimulus alone (Figure 5B, 5C).

### Opposing regulation of *CST5* by SNAIL and VDR activation

**Figure 6:**
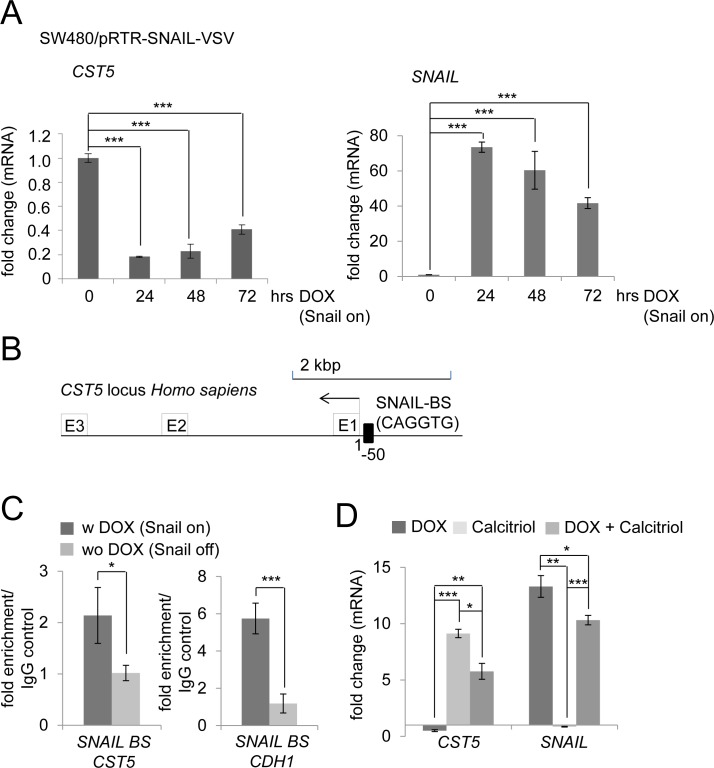
Calcitriol treatment prevents SNAIL-mediated repression of *CST5* **A.** *CST5* and *SNAIL* mRNA levels were measured by qPCR analysis after treatment with DOX at the indicated time-points in SW480/pRTR-SNAIL-VSV cells. Fold changes represent mean values of triplicate analyses after DOX treatment versus vehicle treated cells normalized to *β-actin* expression. **B.** Schematic SNAIL binding site 50 bp upstream of the *CST5* transcriptional start site. **C.** qChIP analysis of DLD1/pRTR-SNAIL-VSV cells 24 hours after activation of SNAIL-VSV expression by addition of DOX using anti-VSV and anti-rabbit IgG for ChIP; this ChIP analysis was performed in unicates and measured in triplicates. **D.** *CST5* and *SNAIL* mRNA levels were measured by qPCR analysis after treatment with DOX, calcitriol or both agents at the indicated time-points in SW480/pRTR-SNAIL-VSV cells. Fold changes represent mean values of triplicate analyses of DOX / calcitriol / DOX+calcitriol treatment versus vehicle treated cells normalized to *β-actin* expression. Error bars represent standard deviations.

Since we had observed that VDR activation by exposure to calcitriol results in the induction of CST5 expression and in the repression of SNAIL, we asked whether conversely SNAIL may directly or indirectly lead to repression of CST5 as part of a SNAIL-induced EMT program. Indeed, *CST5* mRNA levels decreased by up to 80% after induction of *SNAIL* by DOX treatment in SW480/pRTR-SNAIL-VSV cells (Figure 6A). To determine whether this repression is mediated directly by SNAIL occupancy at the *CST5* promoter, we inspected this region for E-boxes, which may mediate SNAIL binding. We identified several E-boxes upstream of the *CST5* promoter and could confirm the direct binding of SNAIL to an E-box, which is located 50 bp upstream of the *CST5* promoter, using qChIP analysis (Figure 6B, 6C). The SNAIL binding site in the *E-cadherin/CDH1* promoter served as a positive control. Next, we assessed the combined effect of SNAIL activation with calcitriol treatment in SW480/pRTR-SNAIL-VSV cells (Figure 6D). When ectopic SNAIL expression was combined with calcitriol treatment, *CST5* was no longer repressed by SNAIL but showed a five-fold induction. Treatment with calcitriol alone led to a robust induction of *CST5,* whereas *SNAIL* mRNA levels were not affected. Therefore, induction of *CST5* expression by VDR activation is dominant over its repression by SNAIL. As discussed below, this may at least partially explain the effect of vitamin D3 in the treatment of cancer.

## DISCUSSION

**Figure 7:**
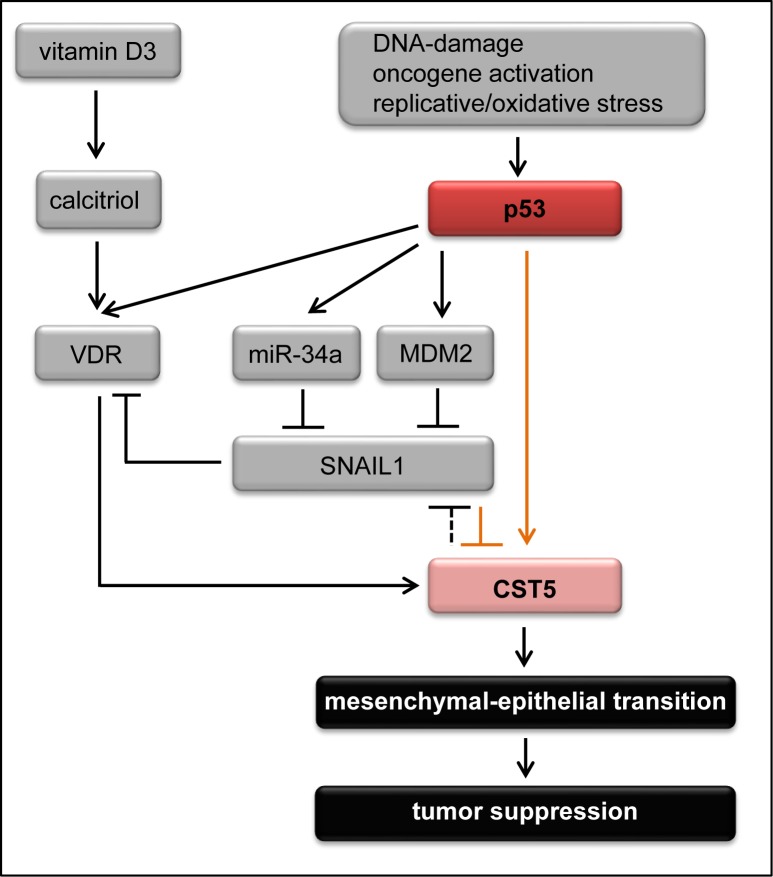
The p53 - vitamin D3 - CST5 regulatory network Schematic model of *CST5* regulation integrating the results of this study (marked with orange arrows) with previous findings (marked with black arrows). Vitamin D3, that is converted to its active metabolite calcitriol, induces *CST5* via the vitamin D receptor (VDR) [25]. In addition, p53 can positively influence *CST5* expression via different pathways. p53 directly induces VDR which might lead to the induction of *CST5* [34]. Furthermore, p53 directly binds to a p53 BS upstream of the *CST5* promoter. And third, p53 can indirectly repress SNAIL expression [65, 66, 70] which results in the induction of *CST5*. Moreover, *CST5* expression indirectly leads to the repression of SNAIL by inhibiting the transcriptional activity of β-catenin/TCF complexes [25]. Upon induction, CST5 promotes mesenchymal-epithelial transition to suppress tumor progression and metastasis.

Here we identified *CST5*, which encodes cystatin D, an inhibitor of cysteine cathepsins, as a new p53 target. Furthermore, we showed that CST5 mediates, at least in part, some of the tumor suppressive functions of p53 in colorectal cancer cells (summarized in Figure 7). We showed that *CST5* is directly induced by p53, which occupies a canonical p53 binding site upstream of the *CST5* promoter. Furthermore, we identified SNAIL as a direct repressor of CST5.

Recently, individual cysteine cathepsins were shown to have diverse roles during tumorigenesis [28, 41]. A study in a mouse model of pancreatic islet cell tumors suggested a multistep process integrating several members of the cysteine cathepsin family that induce angiogenesis, degradation of the basement membrane, tumor growth and invasion [41, 42]. Different members of the cystatin family of protease inhibitors are known to inhibit the protumorigenic actions of cathepsins. For example, cystatin M exerts tumor suppressive effects by inhibiting cell proliferation, migration and invasion in breast cancer cells and is epigenetically silenced in breast cancer [43, 44]. Cystatin D presumably plays an important role as a mediator of tumor suppression in colon cancer cells and is directly induced by the VDR [25] (see also Figure 7). Furthermore, *CST5* expression negatively correlates with human colon cancer progression emphasizing its potential tumor suppressive role for colorectal cancer [25]. By showing that p53 directly induces the expression of *CST5*, we uncovered a new mechanism as to how p53 inhibits tumor progression and growth.

Vitamin D3 is a prohormone, which mainly controls bone mineralization by regulating calcium and phosphate metabolism. During the last years there has been increasing evidence that vitamin D3 deficiency enhances colorectal cancer incidence and mortality [5]. In addition to convincing tumor suppressive effects of vitamin D3 in *in vivo* and *in vitro* studies, meta-analyses of clinical trial results also provided evidence for a tumor suppressive effect of vitamin D3 (reviewed in [1]). Furthermore, several studies showed a negative correlation between 25-hydroxy-vitamin D3 (25(OH)D_3_) serum levels, a marker allowing an overall estimation of the vitamin D3 status, and cancer incidence [45] or overall survival [46], especially for colorectal cancer [20, 47]. Notably, a 30 - 40% reduction of colon cancer risk in patients with high 25(OH)D_3_ serum levels was observed [14]. This is in line with the notion that the risk of colorectal cancer can be strongly influenced by primary prevention including physical activity or diet [48]. Given the strong evidence that vitamin D3 may decrease the risk for colorectal cancer and the world-wide shortage of vitamin D3 alimentation [49], a feasible approach for reducing colorectal cancer incidence and risk would be to increase vitamin D3 uptake by nutritional sources or its synthesis in the skin by exposure to sunlight. Notably, the VDR is highly expressed in early stages of colorectal tumorigenesis but decreases in advanced stages [50-54]. Also p53 mutation is a late event in colorectal cancer development that mostly occurs during the transition from adenoma to carcinoma [55]. Therefore, a combination of p53-activation and calcitriol treatment to enhance *CST5* induction might be more effective in early tumor stages. In case p53 is mutated, a combination of small molecules reactivating mutant p53, such as PRIMA or MIRA-1 [56], together with calcitriol (or an analog to avoid hypercalcemia by high vitamin D3 doses) may lead to the inhibition of cancer progression. Similar approaches combining calcitriol with different known anti-cancer agents, such as doxorubicin or cisplatin, to achieve an enhanced anti-tumor effect have been conducted in several *in vitro* and *in vivo* analyses [57-64]. This provides an opportunity for successful anticancer treatment if chemotherapy alone is not effective. Furthermore, we found that the direct induction of *CST5* by p53 is, at least in part, required for the induction of MET by p53. Several other p53 target genes, among them the *miR-34*, *miR-200* and *miR-15a/16-1* genes, are likely to also contribute to the anti-migratory effects of p53 [65-68].

It has been shown that ectopic expression of CST5 indirectly leads to the repression of SNAIL presumably via the inhibition of the transcriptional activity of β-catenin/TCF complexes [25]. In our study we could show that SNAIL binds upstream of the *CST5* promoter to suppress *CST5* expression and that this repression can be suppressed by treating the cells with calcitriol. It has been shown before that SNAIL can directly repress *VDR* which might also contribute to the inhibition of *CST5* by SNAIL [69]. However, direct binding of SNAIL to the *CST5* promoter has not been demonstrated until now. Since SNAIL-mediated repression of *CST5* is abrogated by calcitriol treatment, calcitriol may inhibit tumor progression even in cells that show high SNAIL expression and therefore have a high tumorigenic potential. Others and we have previously shown that SNAIL expression is downregulated by p53 via the induction of miR-34a [65, 66]. Alternatively, p53 may indirectly suppress SNAIL via MDM2 [70]. Therefore, p53 presumably activates CST5 expression by direct and SNAIL-mediated indirect mechanisms, which together form a feed-forward loop.

Taken together, our findings reveal that *CST5* represents an important mediator of p53 tumor suppression by mediating MET. Notably, *CST5* expression decreases during human colon cancer progression [25]. The possibility to reactivate CST5 expression by dietary vitamin D3 or/and p53 activation suggests that our findings may have potential therapeutic implications.

## MATERIALS AND METHODS

### Cell culture, conditional expression and treatment

The colorectal cancer cell line SW480 was kept in DMEM and the cell lines DLD1, HCT116, and RKO were cultured in McCoys medium supplemented with 10% FCS (Invitrogen) and 1% Penicillin/Streptavidin at 5% CO_2_. For conditional expression of p53, SW480 cells were stably transfected with the episomal expression vector pRTR-p53-VSV [66, 71] or the control vector pRTR using Fugene 6 (Roche) and subsequently selected with 2 μg/ml Puromycin (Sigma; stock solution 2 mg/ml in water) for 10 days. Ectopic expression of p53 was induced for the indicated time-points using 100 ng/ml doxycycline (DOX). HEK293T cells were transfected with the pRTR-p53-VSV vector by calcium-phosphate transfection. siRNA against *CST5* and the respective control (Applied Biosystems) were transfected at a final concentration of 10 nM using HiPerfect (Qiagen). Nutlin-3a (Sigma) and etoposide were both dissolved in DMSO and used at a final concentration of 10 μM and 20 μM, respectively. Calcitriol (Sigma) was dissolved in EtOH and used at a final concentration of 0.1 μM.

### Western blot analysis

SDS-PAGE and Western blotting were performed according to standard protocols. Cells were lysed in RIPA lysis buffer (50 mM Tris/HCl, pH 8.0, 250 mM NaCl, 1% NP40, 0.5% (w/v) Sodium Deoxycholate, 0.1% Sodium Dodecylsulfate, complete mini protease inhibitor tablets (Roche)). Lysates were sonicated and centrifuged at 16.060 g for 15 min, 4°C. 100 μg of whole cell lysate per lane was separated using 10% SDS-Acrylamide gels and transferred on Immobilon PVDF membranes (Millipore). ECL signals were recorded using a CF440 Imager (Kodak). Antibodies used: CST5 (Santa Cruz, sc-46890, 1:200), p21 (Neomarkers, CP-74, 1:1000), SNAIL (R&D Systems, AF3639, 1:250), β-actin (Sigma, A2066, 1:1000).

### Quantitative PCR

Total RNA was isolated using the High Pure RNA Isolation Kit (Roche) according to the manufacturer's instructions. 1 μg of total RNA per sample was used for cDNA-generation using anchored oligo(dT) primers (Ambion Verso Kit). Quantitative PCR (qPCR) was performed by using the Fast SYBR Green Master Mix (Applied Biosystems) and the LightCycler 480 (Roche). Only oligonucleotide pairs resulting in a single peak in the melting curve analysis were used. For sequences of the oligonucleotides used for qPCR see Table 1.

**Table 1:** Oligonucleotides used for qPCR

Gene	Fwd 5′-3′	Rev 5′-3′
*CST5*	CCTCTGCAGGTGATGGCTG [25]	GGACTTGGTGCATGTGGTTC [25]
*p21*	GGCGGCAGACCAGCATGACAGATT	GCAGGGGGCGGCCAGGGTAT
*SNAIL*	GCACATCCGAAGCCACAC	GGAGAAGGTCCGAGCACA
*VDR*	TTGCCATACTGCTGGACGC [69]	GGCTCCCTCCACCATCATT [69]
*β-Actin*	TGACATTAAGGAGAAGCTGTGCTAC	GAGTTGAAGGTAGTTTCGTGGATG

### Immunofluorescence and confocal laser-scanning microscopy

For immunofluorescence analysis SW480/pRTR-p53-VSV cells treated with and without DOX were cultivated on glass cover-slides and fixed in 4% paraformaldehyde/PBS for 10 minutes, permeabilized in 0.2% Triton X 100 for 20 minutes and blocked with 100% FBS for 30 minutes. CST5 (Santa Cruz, sc-46890, dilution 1:50) was used as a primary antibody. α-goat Cy3 (Jackson ImmunoResearch, dilution 1:400) served as secondary antibody. Slides were covered with ProLong Gold antifade (Invitrogen). All stainings were performed without primary antibody as a negative control.

LSM (laser scanning microscopy) images were captured with a confocal microscope (LSM 700, Zeiss) using a Plan Apochromat 20x/0.8 M27 objective, ZEN 2009 software (Zeiss) and the following settings: image size 2048×2048 and 16 bit; Pixel/dwell of 25.2 μs; Pixel Size 0.31 μm; laser power 2%; Master gain 600-1000.

### Wound healing assay

SW480/pRTR-p53-VSV cells were seeded into culture inserts (Ibidi, 80241) and transfected with a siRNA against *CST5* or the respective control for 48 hours. After 42 hours, p53 expression was induced by adding DOX to the indicated samples. Mitomycin C [10 ng/ml] was added for 2 hours before the scratch was applied. To remove mitomycin C and detached cells, cells were washed twice in HBSS +/+ and medium with or without DOX was added. Cells were allowed to close the wound for 48 hours and images were captured on an Axiovert Observer Z.1 microscope connected to an AxioCam MRm camera using the Axiovision software (Zeiss).

### Migration analysis in boyden-chambers

SW480/pRTR-p53-VSV cells were seeded in triplicates into 6-well plates and transfected with siRNA against *CST5* or the respective control for 48 hours. For the last 24 hours previous to the analysis, cells were serum deprived (0.1% serum). To analyse migration, 3×10^5^ cells were seeded in the upper chamber of a Boyden Chamber (8 μm pore size; Corning) in serum free medium. After 42 hours, p53 expression was induced by adding 100 ng/ml DOX to the indicated samples. 10% FCS was used as a chemo-attractant and placed in the lower chamber. Cultures were maintained for 48 hours, then non-motile cells at the top of the filter were removed and the cells in the bottom chamber were fixed with methanol and stained with DAPI. Cell numbers in three different fields per condition were enumerated by immunofluorescence microscopy. The average number of cells in three fields per membrane was counted in triplicates. The relative migration is expressed as the value of treated cells to control cells with the control set as one.

### Chromatin immunoprecipitation (ChIP) assay

SW480/pRTR-p53-VSV, DLD1/pRTR-SNAIL-VSV and HCT116 cells were cultured as described above. Before cross-linking, SW480/pRTR-p53-VSV and DLD1/pRTR-SNAIL-VSV cells were treated with DOX for 24 hours to induce ectopic expression of p53 or SNAIL, respectively, and HCT116 cells were treated with etoposide for 16 hours to induce endogenous p53 expression. Cross-linking was performed with formaldehyde (Merck) at a final concentration of 1% and terminated for SW480/pRTR-p53-VSV and DLD1/pRTR-SNAIL-VSV after five minutes, for HCT116 cells after ten minutes by addition of glycine at a final concentration of 0.125 M. Cells were harvested using SDS buffer (50 mM Tris pH 8.1, 0.5% SDS, 100 mM NaCl, 5 mM EDTA) and after pelleting resuspended in IP buffer (2 parts of SDS buffer and 1 part Triton dilution buffer (100 mM Tris-HCl pH 8.6, 100 mM NaCl, 5 mM EDTA, pH 8.0, 0.2% NaN_3_, 5.0% Triton X-100)). Chromatin was sheared by sonication (HTU SONI 130, G. Heinemann) to generate DNA fragments with an average size of 500 bp. Preclearing and incubation was performed with polyclonal VSV (V4888, Sigma) antibody or IgG control (M-7023, Sigma) using A sepharose beads for SW480/pRTR-p53-VSV and DLD1/pRTR-SNAIL-VSV cells for 16 hours as previously described [72]. The monoclonal DO-1 antibody (Santa Cruz, sc-126) in combination with sepharose G beads was used for the HCT116 cells. Washing and reversal of cross-linking was performed as described in [73]. Immunoprecipitated DNA was analyzed by qRT-PCR. For oligonucleotides used for qChIP see Table 2.

**Table 2:** Oligonucleotides used for qChIP

	Fwd 5′-3′	Rev 5′-3′
CST5 p53 BS	TAAGAGACCGGAAAGGTTGAGA	AGGGCCTTTGCACTGACTATT
CST5 SNAIL BS	GGGGACACCCAAGTAGGATAA	GGAGCTGGATCTCCCAGAG
CDH1 SNAIL BS	TAGAGGGTCACCGCGTCTAT	TCACAGGTGCTTTGCAGTTC
ELF1α	CACACGGCTCACATTGCAT	CACGAACAGCAAAGCGACC

BS: binding site

### ChIP-sequencing

Chromatin immunoprecipitation was performed as described above. The immunoprecipitated DNA-fragments were quantified using a Bioanalyzer (Agilent Technologies). Libraries were generated using a ChIP-Seq Sample Prep Kit (Illumina Part # 11257047) according to the manufacturer's instructions and sequenced on a HiSeq 2000 device (Illumina). 101-nt sequence reads were aligned to the hg19 genome assembly. The complete ChIP-Seq results analysis will be published elsewhere (Hünten et al., submitted).

### Statistical analysis

A Student's *t*-test (unpaired, two-tailed) was used to determine significant differences between two groups of samples. *p*-values < 0.05 were considered as significant (*: *p* < 0.05; **: *p* < 0.01; ***: *p* < 0.001).
